# Rifapentine and isoniazid for prevention of tuberculosis in people with diabetes (PROTID): protocol for a randomised controlled trial

**DOI:** 10.1186/s13063-022-06296-8

**Published:** 2022-06-10

**Authors:** Nyanda Elias Ntinginya, Lindsey te Brake, Issa Sabi, Nyasatu Chamba, Kajiru Kilonzo, Sweetness Laizer, Irene Andia-Biraro, Davis Kibirige, Andrew Peter Kyazze, Sandra Ninsiima, Julia A. Critchley, Renee Romeo, Josephine van de Maat, Willyhelmina Olomi, Lucy Mrema, David Magombola, Issakwisa Habakkuk Mwayula, Katrina Sharples, Philip C. Hill, Reinout van Crevel, On behalf of the PROTID Consortium

**Affiliations:** 1 National Institute for Medical Research (NIMR), Mbeya Medical Research Centre, Mbeya, Tanzania; 2 Departmentt of Pharmacy, Radboud Institute for Health Sciences, Radboud University Medical Center (RUMC), Nijmegen, The Netherlands; 3 The Good Samaritan Foundation (Kilimanjaro Christian Medical Centre GSF KCMC), Moshi, Tanzania; 4 Kilimanjaro Christian Medical University College, Moshi, Tanzania; 5 Department of Internal Medicine, School of Medicine, College of Health Sciences, Makerere University, Kampala, Uganda; 6 Uganda Martyrs Hospital Lubaga, Kampala, Uganda; 7 St George’s University of London, London, UK; 8 King’s College London, London, UK; 9 Department of Internal Medicine and Radboud Center for Infectious Diseases, Radboud University Medical Center, Nijmegen, The Netherlands; 10 Mbeya Zonal Referral Hospital (MZRH), Mbeya, Tanzania; 11 Otago Global Health Institute, University of Otago, Dunedin, New Zealand; 12 Centre for Tropical Medicine and Global Health, Nuffield Department of Medicine, University of Oxford, Oxford, UK

**Keywords:** Preventive treatment, Rifapentine, Isoniazid, Latent tuberculosis infection, Diabetes mellitus

## Abstract

**Background:**

Diabetes mellitus (DM) increases the risk of tuberculosis (TB) and will hamper global TB control due to the dramatic rise in type 2 DM in TB-endemic settings. In this trial, we will examine the efficacy and safety of TB preventive therapy against the development of TB disease in people with DM who have latent TB infection (LTBI), with a 12-week course of rifapentine and isoniazid (3HP).

**Methods:**

The ‘Prevention of tuberculosis in diabetes mellitus’ (PROTID) consortium will randomise 3000 HIV-negative eligible adults with DM and LTBI, as evidenced by a positive tuberculin skin test or interferon gamma release assay, to 12 weeks of 3HP or placebo. Participants will be recruited through screening adult patients attending DM clinics at referral hospitals in Tanzania and Uganda. Patients with previous TB disease or treatment with a rifamycin medication or isoniazid (INH) in the previous 2 years will be excluded. The primary outcome is the occurrence of definite or probable TB disease; secondary outcome measures include adverse events, all-cause mortality and treatment completion. The primary efficacy analysis will be intention-to-treat; per-protocol analyses will also be carried out. We will estimate the ratio of TB incidence rates in intervention and control groups, adjusting for the study site using Poisson regression. Results will be reported as efficacy estimates (1-rate ratio). Cumulative incidence rates allowing for death as a competing risk will also be reported. Approximately 1000 LTBI-negative, HIV-negative participants will be enrolled consecutively into a parallel cohort study to compare the incidence of TB in people with DM who are LTBI negative vs positive. A number of sub-studies will be conducted among others to examine the prevalence of LTBI and active TB, estimate the population impact and cost-effectiveness of LTBI treatment in people living with DM in these African countries and address gaps in the prevention and therapeutic management of combined TB-DM.

**Discussion:**

PROTID is anticipated to generate key evidence to guide decisions over the use of TB preventive treatment among people with DM as an important target group for better global TB control.

**Trial registration:**

ClinicalTrials.govNCT04600167. Registered on 23 October 2020

## Administrative information

Note: the numbers in curly brackets in this protocol refer to SPIRIT checklist item numbers. The order of the items has been modified to group similar items (see http://www.equator-network.org/reporting-guidelines/spirit-2013-statement-defining-standard-protocol-items-for-clinical-trials/).

**Table:** 

Title{1}	Rifapentine and isoniazid for prevention of tuberculosis in people with diabetes (PROTID): protocol for a randomised controlled trial
Trial registration {2a and 2b}	The study protocol has been registered in the clinicaltrials.gov identified with NCT04600167 since 23 Oct 2020
Protocol version{3}	Protocol Version 1.1 dated 25 March 2021 Protocol Number: NIMR-MB-002
Funding {4}	This project is part of the European and Developing Countries Clinical Trials Partnership (EDCTP) 2 programme supported by the European Union (grant number RIA2018CO-2514-PROTID).
Author details{5a}	^*¤^ Contributed equally to this publication ^1^National Institute for Medical Research (NIMR), Mbeya Medical Research Centre, Tanzania ^2^Dept of Pharmacy, Radboud Institute for Health Sciences, Radboud University Medical Center (RUMC), The Netherlands ^3^The Good Samaritan Foundation (Kilimanjaro Christian Medical Centre GSF KCMC), Tanzania ^4^Kilimanjaro Christian Medical University College ^5^Department of Internal Medicine, School of Medicine, College of Health Sciences, Makerere University, Uganda ^6^Uganda Martyrs Hospital Lubaga, Uganda ^7^St George’s University of London, United Kingdom ^8^King’s College London, United Kingdom ^9^Department of Internal Medicine and Radboud Center for Infectious Diseases, Radboud University Medical Center, Nijmegen, The Netherlands ^10^Mbeya Zonal Referral Hospital (MZRH), Tanzania ^11^Otago Global Health Institute, University of Otago, New Zealand ^12^Centre for Tropical Medicine and Global Health, Nuffield Department of Medicine, University of Oxford, Oxford, UK **Corresponding Author:** Dr Nyanda Elias Ntinginya; National Institute for Medical research (NIMR)-Mbeya Medical Research Centre**;** nelias@nimr-mmrc.org
Name and contact information for the trial sponsor {5b}	National Institute for Medical research (NIMR)-Mbeya Medical Research Centre (MMRC) Hospital Hill Rd, P.O.Box 2410 Mbeya Tanzania Tel: +255 25 250 3364
Role of sponsor {5c}	The sponsor is involved in the study design; collection, management, analysis, and interpretation of data; writing of the report; or the decision to publish. The funder (EDCTP ) was not involved in the study design; collection, management, analysis, and interpretation of data; writing of the report; or the decision to publish

## Introduction

### Background and rationale {6a}

Tuberculosis (TB) affects almost 10 million people annually [1]. In addition, it is estimated that one-quarter of the world’s population have latent TB infection (LTBI), of whom 5–10% will go on to develop TB at some stage in their lifetime [2–4]. It is now recognised that the vast global reservoir of LTBI needs to be addressed for there to be any chance of TB elimination [4]. Preventive treatment has up to 90% efficacy against the development of TB in those with LTBI [5]. Certain high-risk groups, such as children in contact with a highly infective TB case and people living with HIV, have already been targeted worldwide for preventive treatment of LTBI [2]. While there are significant operational challenges to consider [6], the clear direction for global TB control is for preventive treatment expansion. The obvious next step is to target other high-risk groups for preventive treatment of LTBI.

One such group is people with DM in TB-endemic countries. People with DM are approximately 3 times more likely to develop TB [7, 8] than people without DM, and experience worse TB treatment outcomes [9]. Globally, about 537 million people live with DM, a number expected to grow to 783 million by the year 2045, by which time as high as 80% of adult DM cases will reside in low- or middle-income countries (LMIC) as they undergo the epidemiologic transition to increasing rates of chronic diseases [10]. Furthermore, the African region is predicted to observe the highest rise in DM burden compared with other regions (IBID). It is estimated that DM now accounts for 15–30% of TB cases globally [11, 12], and this will increase significantly in the coming decades due to the dramatic rise in type 2 DM in TB-endemic settings [12]. Globally, the number of patients with combined TB and DM now already outnumbers that of combined TB and HIV [13, 14].

In a study in Indonesia, TB incidence was higher among people with DM and LTBI (17.1; 95% CI 7.4–33.7/1000 person-years) than those with DM but without LTBI (4.8; 95% CI 0.9–14.0), with an incidence rate ratio of 3.8 (95% CI 0.86 to 20.92; *p* = 0.054) [15]. This high incidence suggests that people with diabetes should be targeted with TB preventive therapy. However, in the absence of clinical trial data, guidelines do not recommend LTBI screening and TB preventive therapy among people with DM.

While INH was the cornerstone of treatment for LTBI for more than 30 years [16, 17], several alternative shortened courses of LTBI treatment have been evaluated more recently, with good safety profiles and treatment courses as short as once daily for 1 month or once weekly for 12 weeks [18–20]. Since 2011, the CDC has recommended the use of 3 months of weekly rifapentine and isoniazid (3HP). A systematic review and meta-analysis including 15 studies of 3HP revealed that this regimen is as safe and effective as other recommended LTBI regimens and achieves significantly higher treatment completion rates [21]. Results are also positive among HIV/TB-coinfected patients [22]. Completion of 3HP in routine healthcare settings was even greater overall than those reported from clinical trials and greater than historically observed using other regimens among reportedly non-adherent populations [23]. However, to date, no RCTs have been conducted to investigate the efficacy and safety of preventive treatment of LTBI in people with DM.

### Objectives {7}

The primary objective of this study is to assess the efficacy of preventive therapy with a 12-week course of rifapentine and isoniazid (3HP) against the development of probable or definite TB disease (see Table 1) over 24 months in people with DM who are LTBI test positive.

**Table 1:** TB diagnostic criteria

Symptom and x-ray result	Investigation results	Definition
**Symptom positive and typical TB on x-ray**	Sputum culture or Xpert positive	Definite TB
	Sputum smear positive, culture negative/not available and Xpert negative/not available	Probable TB
	Sputum smear negative, culture negative/not available and Xpert negative/not available	Possible TB
**Symptom positive, possible TB on x-ray (including extra-pulmonary findings)**	Sputum culture or Xpert positive	Definite TB
	Sputum smear positive, culture negative/not available and Xpert negative/not available	Probable TB
	Sputum smear negative, culture negative/not available and Xpert negative/not available	Possible TB
	Histopathology positive, culture negative/not available and Xpert negative/not available	Probable TB
	Other sample culture or Xpert positive	Definite TB
**Symptom negative, typical TB on x-ray**	Sputum culture or Xpert positive	Definite TB
	Sputum smear positive, culture negative/not available and Xpert negative/not available	Probable TB
	Sputum smear negative, culture and/or Xpert negative	Possible TB
**Symptom positive, normal x-ray**	Sputum culture positive	Definite TB
	Sputum smear positive, culture negative/not available and Xpert negative/not available	Possible TB
	Sputum smear negative, culture negative/not available and Xpert negative/ not available	Not TB
	Histopathology positive, culture negative/not available and Xpert negative/ not available	Probable TB
	Other sample culture or Xpert positive	Definite TB
**Symptom negative, possible TB on x-ray (including extra-pulmonary findings)**	Sputum culture or Xpert positive	Definite TB
	Sputum smear positive, culture negative/not available and Xpert negative/not available	Possible TB
	Sputum smear negative, culture negative/not available and Xpert negative/not available	Possible TB
	Histopathology positive, culture negative/not available and Xpert negative/ not available	Probable TB
	Other sample culture or Xpert positive	Definite TB

^1^A positive GeneXpert is regarded as having the same status as a positive culture result

^2^Symptoms include the clinical sign of adenopathy. ‘Histopathology’ includes microscopy/cell counts of fluid. These definitions regard the initial evaluation. Some patients with possible TB will be put on treatment. A clearcut response to treatment (e.g. improved symptoms and resolution of a pleural effusion) may be considered enough evidence to change a ‘possible’ to a ‘probable’ case

^3^For patients who die without recent TB evaluations, a verbal autopsy will be carried out wherever possible. An endpoint committee will review available data and categorise patients as definite, probable or possible TB; no evidence of TB at death; or unknown TB status at death

Secondary objectives are:

- To assess the efficacy of 3HP against the development of possible, probable or definite TB disease over 24–40 months in people with DM who are latent tuberculosis infection test positive
- To compare the proportions who complete treatment between arms
- To compare the occurrence of adverse events between arms
- To compare the rate of TB or death between arms
- To compare the overall mortality rate between arms
- To explore the efficacy of 3HP against the development of probable or definite TB in those who are LTBI test positive, across the following sub-groups, separately: study site, age groups, duration of DM, level of glycaemic control (baseline HbA1C) and body mass index (BMI)
- To assess the efficacy of 3HP against the development of probable or definite TB, in two restricted analyses: TST-positive and IGRA-positive participants
- To conduct specific sub-studies: economic modelling and cost-effectiveness of treatment, cohort study of LTBI-negative participants, DM phenotype and prevalence of HIV and TB in people with DM, comparison of tests for LTBI and TB, evaluation of point-of-care (POC) testing for LTBI and computer-assisted X-ray; public health study of patient management; and future genetic studies. Details of these sub-studies are not included in this protocol paper.

### Trial design {8}

**Fig. 1:**
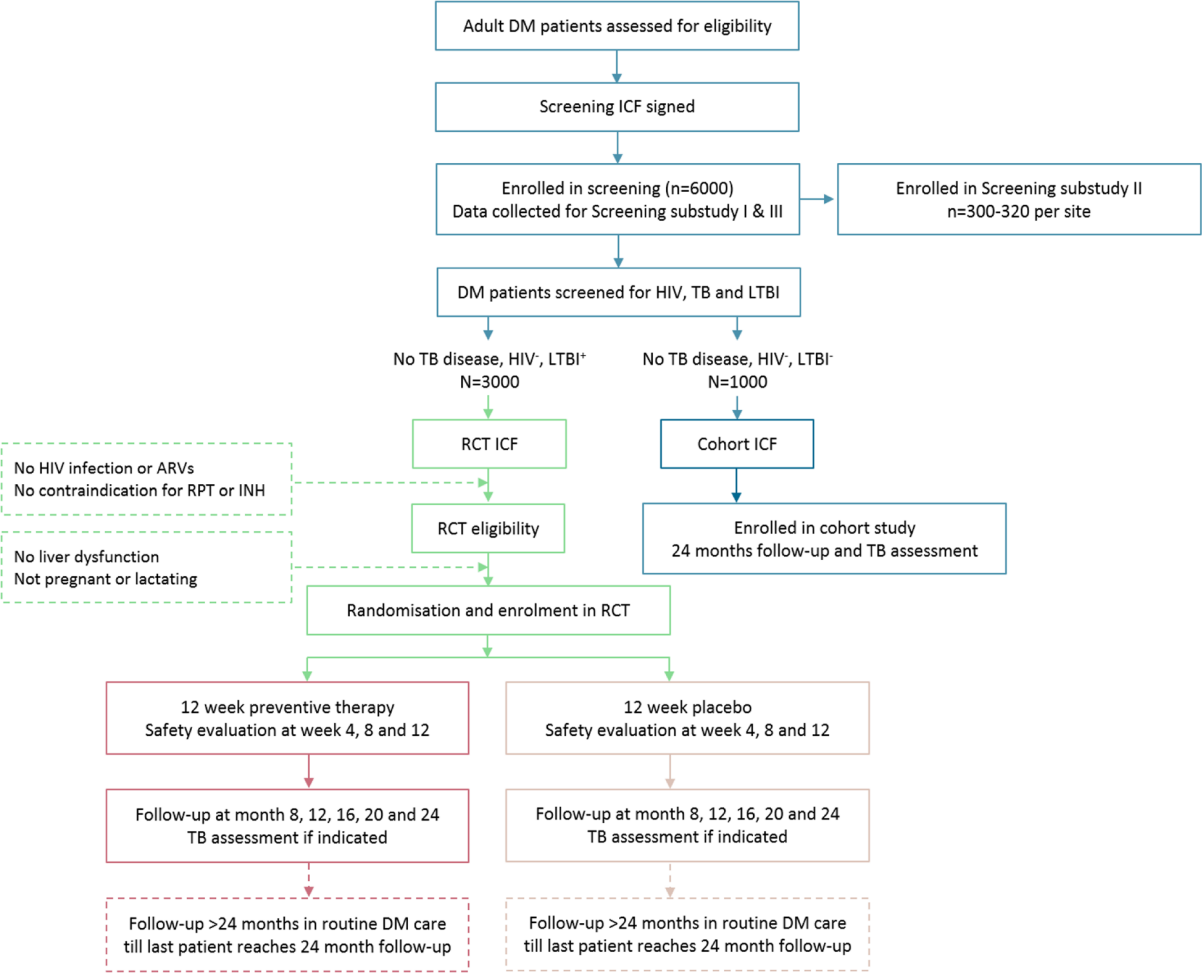
The clinical research flow chart. Explanatory footnotes: The number of participants enrolled in the screening phase (*n* = 6000) is an estimate to yield *n* = 3000 randomised. Screened participants eligible for the RCT or cohort study will be consecutively recruited. Follow-up will consist of 4-monthly scheduled visits for the first 24 months and inter-lock with routine DM clinic care after 24 months. Details of the sub-studies are not provided in this trial protocol paper

This is a multi-site, placebo-controlled, double-blind, individually randomised, phase III efficacy trial. Approximately 6000 adults aged ≥18 years with DM (type 1 or 2) will be tested for LTBI by interferon gamma release assay (IGRA test) and tuberculin skin test (TST). From this, approximately 3000 HIV-negative individuals who are positive on either IGRA or TST will be randomised to receive an oral combination of rifapentine (RPT, 900 mg) and isoniazid (INH, 900 mg) or placebo, once weekly for 12 weeks under direct observed therapy (DOT), and followed up for the development of TB disease and adverse events for at least 24 months (Fig. 1).

## Methods

### Study setting {9}

The study will be conducted in hospitals in Kampala (Uganda) and Moshi and Mbeya (Tanzania).

#### NIMR-Mbeya Medical Research Centre (MMRC), Tanzania, site description

NIMR is the largest public health research institution in Tanzania. NIMR-Mbeya Medical Research Centre (NIMR-MMRC) (http://www.mmrp.org/) is situated on the premises of the Mbeya Zonal Referral Hospital (MZRH), one of the largest referral hospitals serving a population of about 8 million people in seven regions of the southwestern zone of Tanzania and providing a pool for recruitment of participants in various research studies. NIMR-MMRC has conducted high-quality TB and HIV drugs and vaccine trials including EDCTP-funded projects that have contributed into building the capacity and experience to take on the role of sponsor for this study. The project will be recruiting from the internal medicine department of the Mbeya Zonal Referral Hospital with over 1000 DM patients per year. Recruitment could be extended to satellite sites at the Mbeya Regional Referral Hospital (Mbeya), Songwe Regional Referral Hospital (Songwe), and other high-volume health facilities within the catchment area of MZRH.

#### Makerere University, Uganda, site description

Makerere University is one of the oldest and most prestigious universities in Africa. Makerere University will be collaborating with and recruiting from the following hospitals:

Kiruddu National Referral Hospital (KNRH) is a new government-funded and one of three national referral hospitals in Uganda. This study will be carried out at the Diabetic clinic which is managed by the Endocrinology unit with a total of approximately 2500 patients. Uganda Martyrs Hospital, Lubaga, is a missionary hospital situated in Rubaga Division, Kampala City Council Municipality, Uganda, with a total of approximately 1000 DM patients. Kasangati Health centre IV is a public district health facility funded by the Government of Uganda, located in Wakiso district and serving a very large peri-urban and rural population with about 300 DM patients in care.

#### Kilimanjaro Christian Medical Centre (KCMC), Tanzania, site description

KCMC is a referral, consultant, research and teaching hospital located in Northeastern Tanzania. KCMC is a non-profit organization officially opened in 1971 as an institution under the Good Samaritan Foundation (GSF). The hospital caters for the northeastern regions in Tanzania including Kilimanjaro, Arusha, Manyara and Tanga regions and has a catchment population of over 15 million. In response to the growing burden of diabetes, the KCMC diabetic clinic was established in November 1996. The clinic has grown to see approximately 800–1000 diabetes patients per year.

### Eligibility criteria {10}

Participants will be considered eligible for enrolment in the trial if they fulfil all the inclusion criteria and none of the exclusion criteria as defined below.

#### Inclusion criteria

- Enrolled in diabetes care with a history of DM and current use of anti-diabetic medication (‘known DM’); OR in the absence of anti-diabetic medication an HbA1c of ≥6.5% (48 mmol/mol) or a fasting venous plasma glucose of ≥7.0 mmol (126 mg/dl). For those with no previously known DM, a repeat test above the diagnostic cut-point is required to confirm the diagnosis (‘new DM’)
- Adult (18 years or older)
- Diagnosed with LTBI, defined as a positive IGRA test or TST reactivity ≥10 mm
- Voluntarily signed the informed consent form
- If sexually active, willing to use an effective contraceptive method for the duration of preventive therapy

#### Exclusion criteria

- Weight < 45 kg
- Previous TB disease, defined as either bacteriologically confirmed or clinically diagnosed and treated
- Treatment with a rifamycin medication or INH in the previous 2 years
- Diagnosed with TB by a clinician, warranting anti-TB treatment
- Confirmed HIV infection or receiving antiretroviral treatment
- Liver dysfunction, defined as serum aspartate aminotransferase (AST) level 5 times the upper limit of normal
- Pregnant or planning to become pregnant in the next 3 months, or lactating
- Known allergy/sensitivity or any hypersensitivity to components of study drugs or their formulation
- Other conditions inapplicable for participation in this study, such as likely to fail to adhere to study commitment or to complete the whole study, at the discretion of the site investigator
- Participation in another clinical study with an investigational product (IP) or device at the same time

### Who will take informed consent? {26a}

First, people with DM will be asked for written informed consent to participate in the screening phase of the study (Fig. 1). Screening informed consent will include a statement that, pending eligibility, participants may be approached for the trial. Patients potentially eligible for the trial based on screening data will be asked for written informed consent for participation in the trial. Following confirmation of eligibility (i.e. meeting all inclusion/exclusion criteria), eligible patients will enter into the trial and be randomised. Patients eligible for the cohort sub-study will be asked for informed consent for that study separately.

Consent will be in the participant’s own language. Individuals trained and responsible for taking consent (site PI or a designated trained doctor and/or investigator) will be documented on the trial’s Delegation Log. No trial procedures will be performed without informed consent being obtained. It will be made clear in the participant information sheet and verbally that the participant (or their relative) is free to refuse to take part in the study or to withdraw from all or any aspect of the study, at any time and for any reason, without incurring any penalty or affecting their subsequent treatment.

### Additional consent provisions for collection and use of participant data and biological specimens {26b}

The informed consent for the main study will include a statement whereby participants will optionally consent for some of the blood samples taken to be stored for future laboratory sub-studies.

### Interventions

#### Explanation for the choice of comparators {6b}

A no-treatment or non-active comparison group was selected as no treatment is the current standard of care.

#### Intervention description {11a}

Once weekly RPT (6 × 150 mg tablets, Priftin®, Sanofi-Aventis, through the Global Drug Facility) at a fixed dose of 900 mg, combined with once weekly INH (3 × 300 mg tablets, Macleods Pharmaceuticals Limited, through the Global Drug Facility) at a fixed dose of 900 mg, or matching placebo oral tablets. To enable efficient distribution of drugs and trial procedures, drug administration will not be weight-banded. Instead, participants will only be included in the study if they weigh at least 45 kg, to ensure the safe use of fixed doses of 900 mg each of INH and RPT as done by Belknap et al. [24]. Participants will be given the study product as soon after randomisation as possible, and no later than 30 days after screening, defined as all screening and pre-randomisation assessments, is completed. Vitamin B6 daily supplementation to prevent peripheral neuropathy will follow each country’s TB Treatment Guidelines. All participants will continue to receive the standard of care for diabetes according to respective national guidelines, managed by their usual clinical service.

#### Criteria for discontinuing or modifying allocated interventions {11b}

Trial participants may choose to discontinue the trial treatment at any time without penalty or loss of benefits to which they are otherwise entitled. Although the participant is not required to give a reason for discontinuing their trial treatment, a reasonable effort will be made to establish this reason while fully respecting the participant’s rights. Participants who discontinue treatment should remain in the trial for the purpose of follow-up and data analysis (unless the participant withdraws their consent for follow-up).

The following criteria may result in treatment interruption:

- Occurrence of an adverse event
- Requirement for prohibited medications
- Pregnancy or breastfeeding (permanent discontinuation)
- Diagnosed with TB by a clinician, warranting anti-TB treatment (permanent discontinuation)
- Request by the participant to stop treatment
- Intercurrent illness or change in the participant’s condition that justifies the discontinuation of treatment in the treating physician’s opinion and after discussion with the site PI
- Inadequate compliance with the protocol treatment in the judgement of the treating physician

#### Strategies to improve adherence to interventions {11c}

Study drugs will be given orally in the outpatient clinic under the direct supervision of the research staff. Compliance will be confirmed through a review of the medication charts and directly observed administration. Furthermore, participants who miss a visit will be contacted by phone for a maximum of three times after which a maximum of three home visits can be conducted. All contact attempts will be recorded. Participants who miss one or more doses of study treatment can restart within 4 weeks of their last dose and complete their treatment. Subsequent follow-up will be based on their last treatment date.

#### Relevant concomitant care permitted or prohibited during the trial {11d}

All concomitant medications essential for participant management are permitted at enrolment and during the study, except for antiretroviral therapy (see eligibility criteria above). The use of antibiotics with potential anti-TB activity will be monitored, but they will not warrant exclusion from the study. As this is a pragmatic trial, decisions regarding all other medications taken by the participant will be determined by the attending physician according to the clinical circumstances.

RPT is known to induce the hepatic cytochrome CYP450 enzyme system and phase II metabolism. In general, fewer drug-drug interactions are anticipated for 3HP (once weekly) compared to an LTBI regimen with daily rifampicin/RPT. Similar to rifampicin, *daily RPT* significantly reduces plasma exposures of co-administered drugs [25]. In contrast, moxifloxacin exposures remained almost unaffected when RPT was dosed once weekly [26], and RPT given once weekly also did not affect concentrations of efavirenz [27]. Nonetheless, as potential interactions cannot be excluded, the use of these non-study medications (Table 2) during the study phase may result in stopping study treatment when used on a regular basis, as judged by the study physician, and will be monitored at each monthly visit and recorded.

**Table 2:** Relevant concomitant medication prohibited during the trial. Their use should be monitored at each monthly visit during the study treatment up until the first follow-up visit

Medication	
- Methadone - Antibiotics: chloramphenicol, clarithromycin, doxycycline, dapsone - Antifungals: fluconazole, itraconazole, ketoconazole - Anticonvulsants: barbiturates, phenytoin, carbamazepine and sodium valproate - Antiarrhythmics: disopyramide, mexiletine, quinidine, tocainide - Anticoagulant: warfarin - Benzodiazepines: diazepam, lorazepam, etc. - Immunosuppressives: cyclosporine, tacrolimus, corticosteroids - Hormonal contraceptives: Because RPT may compromise the activity of oral contraceptives, a barrier method should be used in addition to oral contraceptives for sexually active female subjects of childbearing age - Haloperidol and newer generation of antipsychotics like risperidone and olazepine - Levothyroxine - Sildenafil (Viagra) - Theophylline - General anaesthetics - Ethanol can exacerbate the potential hepatoxicity of INH and rifamycins. Participants should be urged to abstain from alcohol while on study phase therapy	

The use of the following non-study medications during the study phase will be monitored at each monthly visit and recorded and may result in dose adjustments if given concomitantly with the study drug and pharmacodynamic indices indicate this:

- Antihypertensives: B-blockers, calcium-channel blockers (e.g. verapamil and amlodipine)
- Oral hypoglycaemic agents: sulfonylureas (e.g. glyburide, glimepiride and gliclazide)
- Statins, except rosuvastatin (no interactions with rifamycin anticipated)

### Provisions for post-trial care {30}

Participants will be in the study for a duration of at least 24 months. Follow-up will continue for each participant after 24 months to the end of the trial (i.e. last patient, last visit), with follow-up after 24 months embedded in routine clinical care as per the respective country guidelines. At each post-trial visit, participants will be asked about TB symptoms and other safety outcomes.

### Outcomes {12}

#### Primary outcome

The primary outcome is the occurrence of probable or definite TB disease. Definite TB disease will be diagnosed by a culture or Xpert positive result for *M. tuberculosis.* Probable TB will be diagnosed according to an algorithm that takes into account symptoms, chest x-ray reading, sputum smear, histology and verbal autopsy results (see Table 1).

#### Secondary outcomes

- Occurrence of possible, probable or definite TB disease. We will include those from the algorithm referred to above who are defined as possible TB.
- Occurrence of an adverse event
- Treatment completion, defined as ≥ 11 of 12 doses of treatment over no more than 16 weeks
- All-cause mortality
- Occurrence of possible, probable or definite TB, or death, noting that a proportion of deaths are likely to be due to TB but not possible to confirm through verbal autopsy and clinical notes review.

### Participant timeline {13}

Participants will be followed up in the study for at least 24 months (Fig. 1). Patients will be followed up weekly for the first 12 weeks, then monthly to month 24 (Table 3). Follow-up will continue for each participant after 24 months to the end of the trial, with follow-up after 24 months embedded in routine clinical care as per the respective country guidelines. At each post-trial visit, participants will be asked about TB symptoms and other safety outcomes. Participants may undergo investigations for diagnosis of TB, and other procedures for assessing safety, at any point during follow-up if presenting with symptoms.

**Table 3:** Trial and sub-study assessment schedule for physical visits at study sites

	Screening	Pre-randomisation	Day 0	Week 1	Week 2	Week 3	Week 4	Week 5	Week 6	Week 7	Week 8	Week 9	Week 10	Week 11	Week 12	Month 8	Month 12	Month 16	Month 20	Month 24	Clinic follow-up to end of trial
**Trial eligibility assessment and screening sub-study I: informed consent (screening cohort** ***n*** **= 6000)**	X																				
Demographics, DM and TB (family) history, BMI, blood pressure, waist-hip circumference ^a^	X																				
TST (and reading), IGRA (Quantiferon Gold Plus), HIV	X																				
TB symptoms	X	X		X	X	X	X	X	X	X	X	X	X	X	X	X	X	X	X	X	X^g^
Chest x-ray	X																X			X	
Sputum for Xpert and culture, samples for extra-pulmonary TB, if indicated^b^	X			X	X	X	X	X	X	X	X	X	X	X	X	X	X	X	X	X	X
**Informed consent (trial** ***n*** **= 3000)**		X																			
HbA1c		X															X				
Pregnancy test		X																			
Haematology		X					X				X				X						
Chemistry (eligibility and safety)^c^		X					X				X				X						
Symptoms/adverse events^d^		X		X	X	X	X	X	X	X	X	X	X	X	X	X	X	X	X	X	X^g^
Randomisation			X																		
Dispense IP			X	X	X	X	X	X	X	X	X	X	X	X							
Treatment compliance, discontinuation^e^				X	X	X	X	X	X	X	X	X	X	X	X						
Quality of life^f^		X					X				X				X						
**Cohort study (*n*** **= 1000)**																					
Informed consent for the cohort study		X																			
TST (and reading), Quantiferon Gold Plus		X																			
HbA1c		X																			
TB symptoms		X		X	X	X	X	X	X	X	X	X	X	X	X	X	X	X	X	X	
Chest x-ray																	X			X	
Sputum for Xpert/culture for TB assessment + samples for extra-pulmonary TB if indicated^b^				X	X	X	X	X	X	X	X	X	X	X	X	X	X	X	X	X	
**Blood samples for future laboratory studies**																					
Blood for DNA/RNA/plasma in *n* = 6000 screened and upon TB diagnosis during follow-up (*n* = ~ 100–150)	X															X					
Follow-up Quantiferon Gold Plus in trial (*n* = ~ 600) participants															X						
Blood for DNA/RNA/plasma in trial (*n* = 3000) participants during follow-up															X		X				

^a^Questionnaire (age and sex, education, socioeconomic status, use of alcohol, smoking) and clinical assessment (DM history, DM management, DM complications, TB history, history of exposure to TB, TB treatment, family history of DM and TB, BMI, blood pressure, waist-hip circumference)

^b^Investigations for TB may be carried out at any time if patients present with symptoms of TB. During the screening phase, only Xpert will be used to diagnose TB; the sample will be stored for culture. Investigations for extra-pulmonary TB will be carried out if clinically indicated according to the specified SOP ‘Handling suspected extra-pulmonary TB’

^c^Total bilirubin, AST (SGOT), ALP and Cr

^d^At baseline, all signs and symptoms that occurred within 30 days before entry will be recorded

^e^Doses must be given at least 72 h apart. Adherence will be recorded each week until the completion of treatment (or discontinuation of treatment). Allowable windows are ±2 days for weekly follow-up visits during the 12 weeks of drug administration

^f^Measured using the EQ-5D-5L as part of the cost-effectiveness sub-study

^g^Telephonic follow-up assessment schedule at months 5, 7,10, 14, 18 and 22

Lipid profile (cholesterol, triglycerides, LDL, HDL)

### Sample size {14}

We expect to screen approximately 6000 individuals in order to recruit 3000 patients with DM and LTBI for the RCT. In an unpublished case-control study (excluding HIV patients) in Uganda, the prevalence of LTBI among the DM patients as well as controls was ~ 70% using Quantiferon only.

With an incidence rate of 22.5 TB cases/1000/year, we will have 90% power to detect a RR of 0.5 or less with 3000 individuals randomised (allowing for 10% loss to follow-up). Sensitivity analyses to assess the power of our study under a range of plausible parameter estimates are shown in Table 4. Incidence rates of TB were estimated from prevalence studies, assuming an average disease duration of 2 years. With respect to efficacy, no data are available in DM patients, but a Cochrane Review of 12 trials with 8578 HIV-infected participants reported TB preventive treatment (any anti-TB drug) had a lower incidence of active TB than placebo (RR 0.68, 95% CI 0.54–0.85) [32]. The benefit was greater in TST-positive individuals (RR 0.38, 95% CI 0.25–0.57) than TST-negative individuals (RR 0.89, 95% CI 0.64–1.24) [28]. Risk reduction is expected to be at least as great for DM, and to justify policy change, we envisage that at least 50% efficacy would be needed. TB incidence is expected to stay relatively constant in people with DM (as opposed to the decreasing incidence observed in TB case contacts) which means it is worthwhile to continue follow-up for all trial participants until the last randomised participant completes 24 months of follow-up, in order to maximise power.

**Table 4:** The total number of people needed (with 1:1 randomisation) for detecting a given reduction in risk with 90% power and a significance level of 0.025 (one-sided)

	Cumulative number of cases/1000 over 2 years of follow-up		
RR	30/1000	40/1000	50/1000
0.4	2664	1982	1574
0.5	4106	3054	2424
0.6	6834	5082	4032

### Recruitment {15}

Potential adult participants over 18 years old from hospital with DM outpatient clinics will be identified (~ 6000 in total) and approached by trained clinic staff to participate in the trial. Recruitment activities will not extend to inpatients. The target sample size will be enrolled within an anticipated accrual period of 18 months. All participants with DM will be identified for the screening phase by research staff at the participating sites.

### Assignment of interventions: allocation

#### Sequence generation {16a}

Participants will be randomised 1:1 to intervention or placebo using blocked randomisation within strata (clinical sites), with random block sizes. The randomisation list will be generated and managed by a validated module in the CASTOR database.

#### Concealment mechanism {16b}

Online randomisation will be performed in CASTOR (https://www.castoredc.com). Allocation will be concealed from all involved investigators to ensure blinding to treatment, except for the trial site pharmacists. The treatment allocation for the patient can only be viewed by those with ‘randomisation viewing rights’.

#### Implementation {16c}

A study doctor/nurse will enrol participants and do the online randomisation, which will take place after informed consent has been given, baseline data are complete, and the participant is ready to receive treatment. Patient details will be checked for completeness before randomisation. The website will then provide the treatment allocation for the patient to the site pharmacist who will afterwards prepare the allocated intervention. Blinded drug packages (in fully made-up and labelled treatment packs) with matching placebo tablets will be dispensed.

### Assignment of interventions: blinding

#### Who will be blinded {17a}

All trial staff including the care providers will be blinded except the site pharmacist.

#### Procedure for unblinding if needed {17b}

If, in the opinion of the local clinician, it is crucial for clinical care to unblind treatment (for instance because of a severe reaction that may have other causes that require investigation), the documented request will be discussed with the site principal investigator (PI) and then the chief investigator (CI). The protocol for emergency unblinding (described in an ‘Emergency unblinding’ SOP) includes both secure access to the allocation from the study pharmacist, for the patient’s clinical management team, and clear record keeping of the event in question.

### Data collection and management

#### Plans for assessment and collection of outcomes {18a}

After the written informed consent is obtained from each participant, baseline, outcome and other trial data will be collected on the paper source document and then entered into the database via an electronic case report form (eCRF). All information from a performed visit will be entered into the eCRF in a timely manner (e.g. within 1 week of data generation). Source documents include, but are not limited to, hospital records (from which medical history and previous and concurrent medication may be summarised into the CRF), clinical and office charts, laboratory and pharmacy records, radiographs and correspondence. CRF entries will be considered source data if the CRF is the site of the original recording.

#### Plans to promote participant retention and complete follow-up {18b}

Where possible, study follow-up visits are timed to coincide with routine clinical appointments. Allowable windows are ±2 days for weekly follow-up visits and ± 1 month for 4-monthly follow-up visits to provide some degree of flexibility. Participants who miss a visit will be contacted by phone for a maximum of three times after which a maximum of three home visits can be conducted. All contact attempts will be recorded.

### Data management {19}

All data will be entered by site staff into a cloud-based data management system, CASTOR (https://www.castoredc.com). CASTOR is secured according to the most recent standards and is certified for ISO 27001 (Standards for Information Security Assurance). The data system includes password protection and internal quality checks, such as automatic range checks, to identify data that appear inconsistent, incomplete or inaccurate. Access to data will be determined by the study administrator and can be granted per person per institute.

### Confidentiality {27}

Data will be collected and stored in accordance with GCP and with the EU General Data Protection Regulations.

### Plans for collection, laboratory evaluation and storage of biological specimens for genetic or molecular analysis in this trial/future use {33}

Various biological samples will be collected, evaluated and stored for future laboratory sub-studies as indicated in Table 3.

### Statistical methods

A summary of the statistical approaches is provided here. Full details are outlined in the Statistical Analysis Plan. All efficacy comparisons test hypotheses of the superiority of RPT/INH against placebo, with a significance level of 0.025 (one-sided).

#### Analysis populations

The intention-to-treat (ITT) population, defined as all randomised patients, regardless of whether they are ineligible, withdraw, do not comply with the protocol or do not receive any study treatment, will be the main analysis population. Patients will be analysed according to the intervention they were randomised to receive. The per-protocol population excludes patients who did not complete planned treatment (complete treatment is defined as at least 11 doses of study treatment over no more than 16 weeks) and patients with major protocol violations (eligibility violations, follow-up outside allowable windows and incomplete TB outcome assessment).

#### Statistical methods for primary and secondary outcomes {20a}

The primary efficacy measure will be the ratio of TB (definite or probable) incidence rates (intervention vs control) adjusted for site (the stratification factor) using Poisson regression, reported as an efficacy estimate (1 − rate ratio), with a 95% CI and *p*-value. Deaths with definite or probable TB will be included in the primary endpoint, and only the first TB diagnosis will be included for each participant. For secondary analyses of the primary endpoint, cumulative incidence curves will be fit to estimate cumulative incidence (with 95% confidence bands) accounting for death from non-TB causes as a competing risk. Cox regression analysis will be used to estimate hazard ratios adjusting for age, sex, HbA1c, new vs known DM, body mass index, insulin use, smoking status and recent contact with TB case at baseline.

#### Interim analyses {21b}

We anticipate there will be 2 formal interim efficacy analyses, after 1/3 and 2/3 of the information has accrued; however, the DSMB may alter the timing and number of efficacy analyses as required. The DSMB may recommend early termination of the trial based on these analyses. Such a recommendation would be made if, in the view of the DSMB, there is proof beyond reasonable doubt that the intervention is better than placebo control, or if there were significant safety concerns. The Haybittle-Peto boundary, requiring *p* < 0.001 at an interim analysis to consider stopping for efficacy, will be used as guidance.

Given the lack of data on event rates at the study sites at the time of protocol development, the DSMB will review the event rates before recruitment is completed, to consider the extension of recruitment if event rates are lower than anticipated.

#### Methods for additional analyses (e.g. subgroup analyses) {20b}

Analyses of secondary outcomes of (i) possible, probable or definite TB; (ii) TB incidence of death (all causes); and (iii) overall mortality will follow the same approach as for the primary outcome. For safety and completion of treatment outcome, we will compare the proportions of participants experiencing each event by arm using either *χ*^2^ or Fisher’s exact tests (depending on numbers of events). No formal adjustments are made for multiplicity across the secondary analyses, but results will be interpreted with appropriate caution.

Subgroup analyses will be conducted for the primary outcome, if the overall treatment effect is significant, by including interaction terms in the analyses. The pre-specified subgroup comparisons are area (Mbeya, Moshi, Uganda), age group (approximately tertiles), duration of DM (approximately tertiles), DM status (new vs known), BMI (as a continuous and categorical variable) and level of glycaemic control (HbA1c > 8%) at baseline. In addition, we will repeat the primary analysis (i) restricted to the group who are TST positive and (ii) restricted to the group who are IGRA positive.

#### Methods in analysis to handle protocol non-adherence and any statistical methods to handle missing data {20c}

Per-protocol analysis will be used as a secondary analysis of the primary endpoint. In the per-protocol analysis, only patients who receive the study drug as planned will be analysed; this will exclude patients who did not complete treatment and participants with major protocol violations.

The primary analysis will not adjust for missing data, but multiple imputation will be carried out as a sensitivity analysis to account for data that are missing at random (MAR). In addition, sensitivity analyses will be carried out to evaluate the robustness of the conclusions to a range of plausible patterns of missing not at random (MNAR) using pattern mixture or selection models.

### Plans to give access to the full protocol, participant-level data and statistical code {31c}

The protocol will be readily available; however, corresponding data generated from this trial will not be for the public. The PROTID trial has been registered in the clinicaltrials.gov identified with NCT04600167 since 23 Oct 2020.

## Oversight and monitoring

### Composition of the coordinating centre and trial steering committee {5d}

**Fig. 2:**
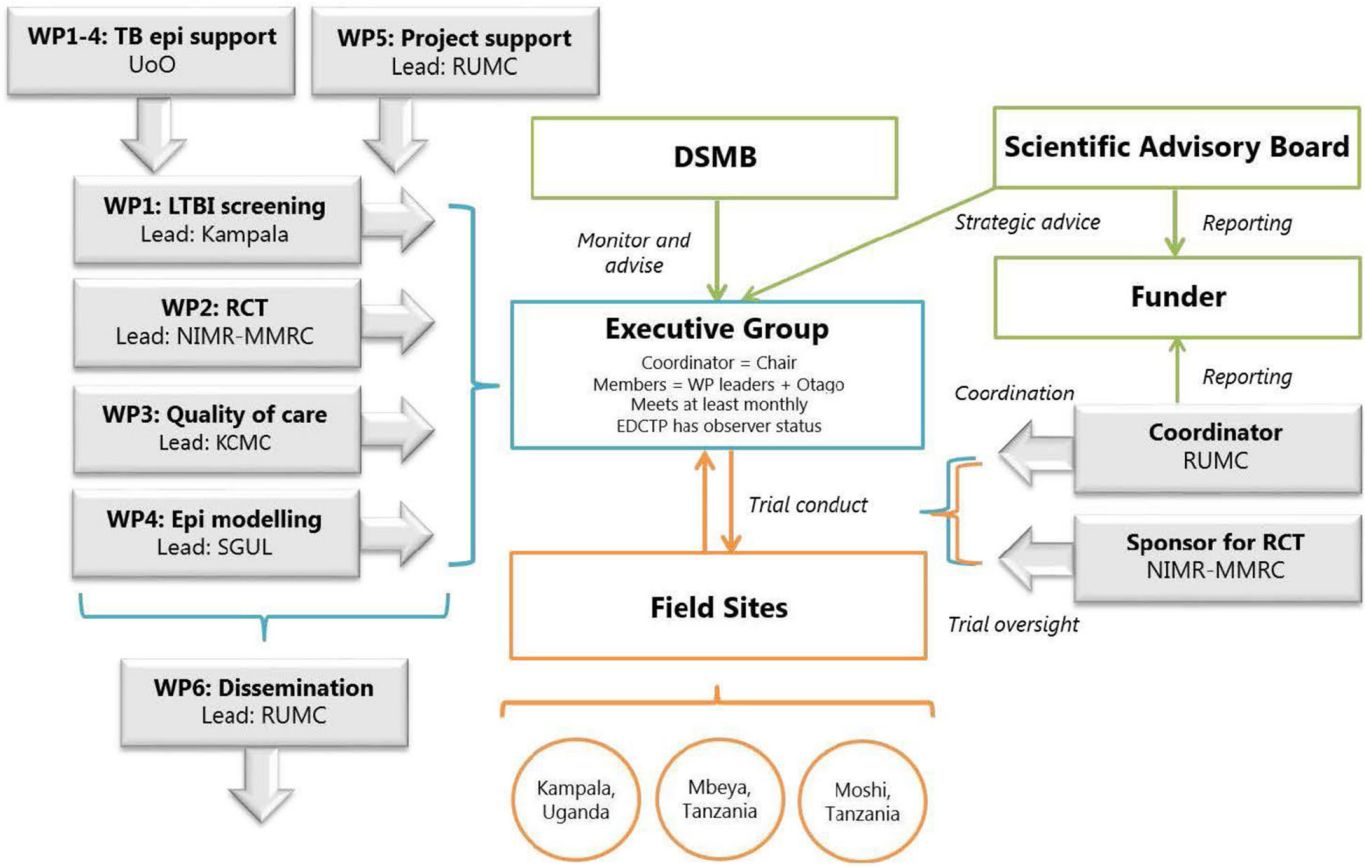
Project organogram with Work Package (WP) 2 representing the PROTID trial

Overall project management and progress monitoring will be performed by the Executive Group (EG). The EG will be led by the project coordinator and the trial responsibility will be with the trial sponsor. Preferably, decisions will be made by consensus; otherwise, the matter will be discussed with the DMC and Scientific Advisory Board (SAB) in order to make a decision if necessary. The day-to-day work of the project will be handled by the field sites that also coordinate local regulatory, diagnostic work and data management. Sites will report progress and receive guidance from the trial sponsor and EG. EG members will meet regularly with a frequency of at least a month, which can be intensified based on the current need. A SAB has been formed to provide independent advice to the trial. The SAB will meet at least annually and discuss overall scientific strategy and progress. In addition, an independent DSMB has been established with its primary responsibility being to safeguard the interests of trial participants by monitoring participant safety, assess participant risk versus benefit and assess data quality. The management approach is described graphically on the project organogram (Fig. 2).

### Composition of the data monitoring committee, its role and reporting structure {21a}

The DSMB is composed of five members, with clinical and methodological expertise, including an independent statistician. The DSMB will review results at regular intervals, blinded to treatment allocation in the first instance, but may be unblinded if deemed necessary in consultation with the project EG. Access to interim data and results on study safety or efficacy will be confidential and strictly limited to the DSMB.

### Adverse event reporting and harms {22}

(Serious) adverse event ((S)AE) definitions are in line with GCP guidelines. In the context of this trial, AEs include an exacerbation of a pre-existing illness, an increase in frequency or intensity of a pre-existing episodic event or condition, a condition (even though it may have been present prior to the start of the trial) detected after trial drug administration, and continuous persistent disease or a symptom present at baseline that worsens following administration of the study treatment. AEs do not include medical or surgical procedures—the condition that leads to the procedure is the adverse event; hospitalizations where no untoward or unintended response has occurred, e.g. elective cosmetic surgery or social admissions. Death will always be reported as a serious adverse event, regardless of cause. The AE recording and reporting period for this study are from study enrolment until the end of the trial. At all clinical assessments, including scheduled and unscheduled, information on AEs will be elicited through consultation with the participant. AE information will also be collected through consultation with the participant’s medical team and from their medical records. Blood tests additional to those described in the trial schedule may be requested at any time for clinical management of the participant. The SAE reporting period is from randomisation to 60 days after the end of study treatment. (S)AEs will be managed in all participants according to standard clinical practice in line with trial AE management procedures. All (S)AEs will be reported on CRFs, coded centrally at the sponsor site according to event type and severity using the Common Toxicity Criteria for Adverse Events (CTCAE) grading scale v5.0. Deaths will be coded to CTCAE where possible based on verbal autopsy and clinical note review. SAEs will be reported to the sponsor and the DSMB within 24 h of the site becoming aware of the event using SAE report forms.

### Frequency and plans for auditing trial conduct {23}

On-site monitoring will be regularly conducted by the internal site monitors complemented by external monitors from Clinical Research Organization (CRO). The frequency, type and intensity of routine monitoring and the requirements for triggered monitoring are detailed in the Monitoring Plan which also details the procedures for review and sign-off. The monitoring will adhere to the principles of GCP.

### Plans for communicating important protocol amendments to relevant parties (e.g. trial participants, ethical committees) {25}

Changes to the protocol require the EG to seek permission from the ethics and regulatory authorities as guided by respective regulations in Tanzania and Uganda.

### Dissemination plans {31a}

We will develop a publication and dissemination plan to include conference presentation(s) and journal publication(s). We also plan dissemination to relevant patient and clinical interest groups as well as the responsible ministries. Any publication arising before all patients have completed ≤24 months of follow-up will also be approved by the DSMB in order to ensure that the primary objective of the trial is not compromised.

## Discussion

The PROTID trial addresses a prevention priority area (TB and DM comorbidity) and is anticipated to generate the first solid evidence with knowledge and practical applications to support or refute the use of preventive treatment against TB among people with DM. If successful, the trial will contribute to improve the management and outcome of combined TB and DM.

Several challenges and risks can be foreseen; the first entails the global shortage of RPT, for which we have been in close contact with collaborators at the KNCV TB foundation and the Global Drug Facility. Second, the discolouration caused by rifamycins of bodily fluids (yellow, orange, red or brown) may compromise blinding procedures of this trial. Third, the COVID-19 pandemic may cause unforeseen and significant disruption of societal and health system functioning. Areas of particular concern include DM treatment continuity, supply chain management and TB/COVID-19 diagnosis. Fourth, there are potential drug-drug interactions caused by once weekly RPT. Relatively limited data are available on the impact of once weekly RPT on co-administered medications. We hypothesise that the impact will be limited as compared to a daily rifamycin regimen, but we will strictly record all concomitant medication in this study with special attention to the effectiveness of co-administered antihypertensives, oral hypoglycemic agents and statins.

This study will be conducted in Uganda and Tanzania, East Africa, a region that is expected to have an increase of DM prevalence [29]. Both countries are endemic for TB and are among the 30 high TB burden countries. They have limited available data on the epidemiology of TB-DM or on DM phenotypes and management within the health systems. Our project therefore will make an important contribution to guide health priority setting at national and international levels in regard to TB-DM management. Our consortium includes partners who have worked together previously and are experienced in performing RCTs in high TB and DM burdens. By creating networking and exchange visits, we are building an excellent platform for future collaborative clinical trials and interlocking studies addressing combined TB and DM as well as other interactions between communicable and non-communicable diseases in sub-Saharan Africa.

## Trial status

Protocol version 1.1 dated 25 March 2021. Protocol number: NIMR-MB-002

The recruitment is expected to begin during April 2022 with a follow-up of at least 24 months from the trial start with the estimated primary completion date in May 2024.

### Authors’ contributions {31b}

PH, RvC, KS, WO, LtB, NEN, JC, KK, IAB, DK and NC conceived the study in the protocol paper; NEN and LtB drafted the first version of the manuscript; KS, PH and RvC made substantial revisions; KS, PH and WO specifically addressed sections on statistical analysis; all authors reviewed and agreed with the final version.

### Funding {31b}

This project is part of the European and Developing Countries Clinical Trials Partnership (EDCTP) 2 programme supported by the European Union (grant number RIA2018CO-2514-PROTID).

## Abbreviations

3HP: 3-month/3-week course of rifapentine and isoniazid
AEs: Adverse events
ALP: Alkaline phosphatase
AST: Aspartate aminotransferase
BMI: Body mass index
CDC: Center for Disease Control
CI: Confidence interval
CI: Chief investigator
COVID-19: Coronavirus disease of 2019
Cr: Creatinine
CRO: Clinical Research Organization
CTCAE: Common Toxicity Criteria for Adverse Events
CYP450: Cytochrome P450
DM: Diabetes mellitus
DMC: Data Monitoring Committee
DOT: Direct observed therapy
DSMB: Data and Safety Monitoring Board
eCRF: Electronic case report form
EDCTP: European and Developing Countries Clinical Trials Partnership
EG: Executive Group
EQ-5D-5L: 5-Level EQ-5D version
EU: European Union
GCP: Good Clinical Practice
GSF: Good Samaritan Foundation
HbA1c: Glycated haemoglobin
HDL: High-density lipoprotein
HIV: Human immunodeficiency virus
IGRA: Interferon gamma release assay
INH: Isoniazid
IP: Investigational product
ISO: International Organization for Standardization
ITT: Intention-to-treat
KCMC: Kilimanjaro Christian Medical Centre
KNCV TB: KNCV Tuberculosis Foundation
LDL: Low-density lipoprotein
LMIC: Low- or middle-income countries
LTBI: Latent TB infection
MAR: Missing at random
MNRH: Mulago National Referral Hospital
MRCC: Medical Research Coordinating Committee
MZRH: Mbeya Zonal Referral Hospital
NDA: National Drug Authority
NIMR-MMRC: National Institute for Medical Research-Mbeya Medical Research Centre
POC: Point-of-care test
RCTs: Randomised control trials
RPT: Rifapentine
RUMC: Radboud University Medical Center
SAB: Scientific Advisory Board
SAE: Severe advent event
SGOT: Serum glutamate oxalate transaminase
SOP: Standard operation procedure
TB: Tuberculosis
TMDA: Tanzania Medicines and Medical Devices Authority
TST: Tuberculin skin test
WP1: Work Package 1
WP2: Work Package 2
WP3: Work Package 3
WP4: Work Package 4
WP5: Work Package 5
